# Socio-demographic indicators of self-reported health based on EQ-5D-3L: A cross-country analysis of population surveys from 18 countries

**DOI:** 10.3389/fpubh.2022.959252

**Published:** 2023-01-06

**Authors:** Agota Szende, Mathieu F. Janssen, Juan Cabases, Juan M. Ramos-Goni, Kristina Burström

**Affiliations:** ^1^Global Health Economics and Outcomes Research, Labcorp, Leeds, United Kingdom; ^2^Medical Psychology and Psychotherapy, Erasmus MC, Rotterdam, Netherlands; ^3^EuroQol Group, Rotterdam, Netherlands; ^4^Department of Economics, Public University of Navarra, Pamplona, Spain; ^5^Maths in Health B.V., Rotterdam, Netherlands; ^6^Health Outcomes and Economic Evaluation Research Group, Stockholm Centre for Healthcare Ethics, Department of Learning, Informatics, Management, and Ethics, Karolinska Institutet, Stockholm, Sweden; ^7^Equity and Health Policy Research Group, Department of Global Public Health, Karolinska Institutet, Stockholm, Sweden

**Keywords:** EQ-5D-3L, EuroQol, health-related quality of life, self-reported health, population health, health inequalities, equity, sociodemographic indicators

## Abstract

**Background:**

Generic health-related quality of life instruments, such as the EQ-5D, are increasingly used by countries to monitor population health via general population health surveys. Our aim was to demonstrate analytic options to measure socio-demographic differences in self-reported health using the EuroQol Group's archive of EQ-5D-3L population surveys that accumulated over the past two decades.

**Methods:**

Analyses captured self-reported EQ-5D-3L data on over 100,000 individuals from 18 countries with nationally representative population surveys. Socio-demographic indicators employed were age, sex, educational level and income. Logistic regression odds ratios and the health concentration index methodology were used in the socio-demographic analysis of EQ-5D-3L data.

**Results:**

Statistically significant socio-demographic differences existed in all countries (*p* < 0.01) with the EQ VAS based health concentration index varying from 0.090 to 0.157 across countries. Age had generally the largest contributing share, while educational level also had a consistent role in explaining lower levels of self-reported health. Further analysis in a subset of 7 countries with income data showed that, beyond educational level, income itself had an additional significant impact on self-reported health. Among the 5 dimensions of the EQ-5D-3L descriptive system, problems with usual activities and pain/discomfort had the largest contribution to the concentration of overall self-assessed health measured on the EQ VAS in most countries.

**Conclusion:**

The EQ-5D-3L was shown to be a powerful multi-dimensional instrument in the analyses of socio-demographic differences in self-reported health using various analytic methods. It offered a unique insight of inequalities by health dimensions.

## Background

Socio-demographic differences in health have been traditionally investigated using measures of health, such as mortality and morbidity by disease categories in population health monitoring surveys. Self-reported measures have also been included in such surveys but have mostly been limited to one-dimensional self-rated health measures (1, 2). In particular, the lack of standardized, more complex self-reported health indicators in European databases has been noted (3). For example, in a prominent European study, Mackenbach et al. examined socioeconomic inequalities in self-assessed health in 22 countries using the single-item self-rated health question (4). Furthermore, Eurostat still uses single-item categorical measure of self-reported health (5). Some researchers, however, started to apply multi-dimensional health-related quality of life (HRQoL) data in the socio-economic analysis of health, which included generic measures, such as the EQ-5D and SF-36 (6–12). Some countries have also implemented regular monitoring of population health using the EQ-5D instrument in nationally or regionally representative health surveys, such as Canada, Spain, Sweden, and the UK. With the increased use of HRQoL measures, such as the EQ-5D, in monitoring population health, there is a need for understanding options for analyzing socio-demographic differences with such data.

Over the past two decades, the EuroQol Group has accumulated HRQoL data from general population surveys through its network of researchers across multiple countries. A useful feature of the database archive is that it contains standardized HRQoL self-reported data based on the EuroQol Group's widely used generic instrument, the EQ-5D-3L. Respondents can report their health on the 5 dimensions of the EQ-5D-3L descriptive system, including mobility, self-care, usual activities, pain/discomfort, and anxiety/depression. Respondents also rate their overall assessment of their health state today on a visual analog scale (EQ VAS), anchored on a scale 0 (worst imaginable health state) and 100 (best imaginable health state) (13, 14). The EQ VAS measure is therefore closer to the other health indicators like the global rating of health. As such, the EQ-5D database archive offers the opportunity to explore socio-demographic differences both along the 5 dimensions of the EQ-5D-3L descriptive system as well as in self-assessed overall health along the EQ VAS as reported by general populations of 18 countries.

The objective of the present analysis was to study the potential use of the EQ-5D-3L to explore how self-assessed overall health EQ VAS and problems reported along the 5 dimensions of the EQ-5D-3L descriptive system vary by selected socio-demographic indicators. In terms of analytic methods, it is interesting to explore how the EQ-5D-3L can be used with approaches that focus on comparing groups, such as by odds ratios, vs. those that assess the entire population, such as concentration indices.

In addition to the methodological aspects of using the EQ-5D-3L instrument in this area, the aim of this study is also to inform researchers and policy-makers in each country with valuable approaches and past EQ-5D-3L data that they can consider within the light of their own social and health care context and monitor changes that occur in the future.

## Methods

### EQ-5D-3L survey sample

The EQ-5D-3L has been administered in national general population surveys in 18 countries *via* postal and face-to-face surveys between 1993 and 2010 and have been archived by the EuroQol Group (10, 15–28). The characteristics of individual surveys are summarized in Table 1.

**Table 1:** National EQ-5D-3L population surveys in 18 countries.

	**Source**	**Sample size**	**Data collection**
Belgium*	ESEMED, König et al. (16)	2,411	2001-2003
China	Sun et al. (10)	8,031	2010
Denmark	Sorensen et al. (17)	16,861	2000-2001
Finland	Saarni et al. (19)	8,028	1992
France*	ESEMED, König et al. (16)	2,892	2001-2003
Germany*	ESEMED, König et al. (16)	3,552	2001-2003
Greece	Yfantopoulous (20)	464	1998
Hungary	Szende and Nemeth (21)	5,503	2000
Italy*	ESEMED, König et al. (16)	4,709	2001-2003
Korea	Lee et al. (22)	1,307	2007
Netherlands*	ESEMED, König et al. (16)	2,367	2001-2003
New Zealand	Devlin et al. (23)	1,327	1999
Slovenia	Prevolnik and Rebolj (24)	742	2000
Spain*	ESEMED, König et al. (16)	5,473	2001-2003
Sweden	Björk et al. (25)	534	1994
Thailand	Tongsiri et al. (26)	1,409	2007
United Kingdom	Kind et al. (27)	3,395	1993
England	Health Survey for England, (18)	4,645	2010
US*	MEPS, Sullivan et al. (28)	38,678	2000-2002

* Surveys in 7 countries included income data: Belgium, France, Germany, Italy, Netherlands, Spain, and the United States.

Surveys differed in sample sizes and in the mode of administration and time of data collection. The United States dataset had the largest sample with over 38,000 respondents, while the Greek and the Swedish national surveys had the smallest sample of around 500 respondents. Some of the surveys were postal while others were performed as part of a face-to-face interview or administered by telephone. While only the most recent national surveys were included in the analysis from each country, the date of data collection varied considerably across countries. Data collection for the majority of surveys took place during or after 2000, however some surveys were older with the United Kingdom and Swedish datasets being the earliest from 1993 and 1994, respectively. These differences should be considered when interpreting results, given that HRQoL in general and specifically EQ-5D ratings and values could have changed over time. In the United Kingdom a newer representative survey was conducted in 2010 in England but it did not include the EQ VAS, nor did the Finnish dataset. For this reason, indicators that required EQ VAS were reported using the older UK dataset, while these were not reported for Finland.

All surveys captured socio-demographic data - such as age, sex, and educational level - that were included in a standardized format in the dataset archive. Seven surveys out of the 18 countries also captured income data that allowed for separate expanded analyses in a subset of these countries, including Belgium, France, Germany, Italy, Netherlands, Spain, and the United States.

### Variables

EQ-5D-3L variables used in the analysis included both the EQ VAS, and reported problems along the 5 dimensions of the EQ-5D-3L descriptive system. To capture morbidity by a binary measure in general population surveys where the majority of people report no problem, level 2 (some problems) and level 3 (severe problems) were combined to result in one variable for reporting “any” problems.

Socio-demographic indicators captured 4 key variables, including age, sex, education level, and income. The selection of these variables was driven by the availability of socio-demographic data across countries in the data archive. Age was measured as a continuous variable and categorized into 10-year age groups: 18–24; 25–34; 35–44; 45–54; 55–64; 65–74; 75+ (reference group 18–24 years). Sex was recorded as a categorical variable (reference group males). Educational level in each country was categorized into low (equivalent to primary); medium (equivalent to secondary) and high (equivalent to levels above secondary education). Medium and high educational levels were combined into one category and used as reference group. As such, educational level was used as a binary variable to focus on those with the lowest educational level.

Income measurement in the subset of the 7 countries with income data included both continuous and categorical measurements. In the European countries, categorical income data included 4 categories, while the US MEPS dataset included 5 income categories. Categorical income data were used in the odds ratio analyses while continuous income data were used to rank people by income level in the concentration index analyses. In addition, income was defined in two different ways and analyses were repeated for each approach. First, income defined as per capita income was used in the main analysis. Secondly, income was defined as equivalent income based on household income and household composition in the additional analyses. Equivalent income was calculated using the OECD modified scale, which includes the scale of 1, 1.5, 1.8, 2.1, and 2.4 for household size of 1, 2, 3, 4, and 5 members, respectively. The scale aims to reflect economies of scale in consumptions with increasing household size.^1^

### Analytic approaches

Two main analytic approaches were used: odds ratios that focus on comparing groups of the population and concentration indices that assess how unequally health is distributed across the entire population.

Logistic regression age-adjusted odds ratios for reporting problems on each EQ-5D-3L dimension were calculated by age groups, sex, and educational level. An odds ratio higher than 1 indicates that the examined group reported a higher prevalence of health problems than the reference group. In the subset of 7 countries with income data, separate logistic regression odds ratios (adjusted for confounding factors) for reporting problems along the 5 dimensions of the EQ-5D-3L descriptive system were calculated by income groups. In addition, differences in EQ-5D-3L (EQ VAS rating and reported problems along the 5 dimensions of the EQ-5D-3L descriptive system) between the lowest and highest income groups were also calculated.

The concentration index method, which is a single index measure of relative inequalities in health (29, 30), was also used in the analysis. The overall health concentration index measures the mean difference in health between individuals as a proportion of the average health of the total population. This index can be interpreted as a measure of how unequal the distribution of health is in the population. Health inequality is measured on a scale between 0 (defined as complete equality in health) and 1 (defined as complete inequality in health). It has been shown that the concentration index value also corresponds to 75% of the Schutz index, and as such, it can also be interpreted as the proportion of health that should be redistributed from those above the average level to those below the average in order to equalize the distribution of health (31). The concentration index method has been used in previous inequality studies, including surveys of self-assessed health measured on single dimensional scales as well as multi-dimensional measures, such as the EQ-5D-3L in individual country studies (8, 32, 33).

The overall concentration index can be decomposed to identify the impact of various factors, such as socio-demographic indicators or HRQoL characteristics, in order to determine how much each factor contributes to the inequalities (34, 35). In the current analysis, overall self-assessed health measured on the EQ VAS. Decomposition analysis was performed and presented in three models to determine inequalities by socio-demographic indicators, the 5 dimensions of the EQ-5D-3L descriptive system, and in a combined model in which both socio-demographic indicators and the 5 dimensions of the EQ-5D-3L descriptive system were included.

In the subset of the 7 countries with income data, the concentration index analysis also used overall self-assessed health measured on the EQ VAS. However, individuals were ranked by income level and their EQ VAS score was used to determine the overall health concentration index. Decomposition analysis was performed to characterize inequalities along the 5 dimensions of the EQ-5D-3L descriptive system. Variables for the decomposition analysis included the socio-demographic variables age, sex and educational level, and reported problems along the 5 dimensions of the EQ-5D-3L descriptive system.

The concentration indices were computed by the convenient regression model as proposed by Kakwani et al. (29) – see Supplementary material.

Data analyses were performed using SPSS version 19 and Stata version 12 statistical software packages. All codes were checked and analyses were reproduced independently by a second analyst.

## Results

In Table 2, the sample characteristics, mean age, sex distribution, percentage with low educationalal level, and prevalence of reported problems along the 5 dimensions of the EQ-5D-3L descriptive system and mean EQ VAS score are provided by country.

**Table 2:** Summary sample characteristics in 18 countries.

	**Age mean**	**Sex % male**	Education % with low educational level*	**EQVAS (mean value)**	**Mobility∧% of any problems**	**Self-care∧ % of any problems**	**Usual activities∧% of any problems**	**Pain/discomfort∧ % of any problems**	**Anxiety/depression∧% of any problems**
Belgium	48.2	49.3	32.5	77.6	12.6	4.0	12.4	28.5	6.6
China	46.8	48.3	43.5	80.4	5.1	2.8	5.2	10.7	8.7
Denmark	46.5	46.9	48.6	83.7	10.7	2.5	17.9	36.7	16.1
England	35.9	46.4	31.0	NA	19.4	5.6	17.0	35.3	19.3
Finland	54.2	45.3	42.1	NA	26.3	8.6	21.0	47.8	13.9
France	46.8	45.9	65.0	76.8	13.4	4.0	10.0	35.9	15.0
Germany	48.1	46.7	3.3	77.3	15.9	2.7	9.9	27.6	4.3
Greece	42.2	54.1	44.3	79.0	13.3	5.7	10.5	16.8	10.7
Hungary	46.6	44.8	33.4	71.1	19.6	6.5	14.8	39.2	35.2
Italy	46.8	49.2	59.6	77.1	10.4	3.3	9.4	26.6	8.7
Korea	42.7	49.0	19.8	79.5	5.9	0.8	4.1	21.3	17.4
Netherlands	48.4	43.5	36.1	82.0	11.5	3.4	13.5	34.2	3.5
New Zealand	49.9	43.3	27.9	80.8	20.0	4.4	21.5	40.8	21.2
Slovenia	44.2	43.7	14.2	76.4	29.8	14.0	32.9	47.2	36.4
Spain	48.6	44.2	60.1	75.0	13.7	4.1	11.7	22.9	7.8
Sweden	45.7	49.4	28.8	83.3	8.6	1.5	7.9	40.8	26.0
Thailand	44.6	45.7	NA	79.4	26.3	8.7	22.7	65.0	47.4
United Kingdom	47.9	43.3	39.8	82.8	18.4	4.3	16.3	33.0	21.0
United States	45.6	45.8	25.8	80.0	18.5	3.7	17.9	48.3	23.2

* Low educational level (as opposed to medium or high education level). ∧Any problems = some or severe problems. These results reflect the raw total population scores and cannot be compared directly across countries as they reflect the unique age structure within each country. This is in line with the states objective of the analysis not to rank countries based on inequality results.

### Odds ratios for reporting problems on EQ-5D-3L dimensions

Table 3 shows the odds ratios for age (each additional decade of life), sex (women vs. men), and educationalal level (low vs. middle/high) are presented by country and by the 5 dimensions of the EQ-5D-3L descriptive system.

**Table 3:** Odds ratios (95% confidence intervals) for reporting any problems on EQ-5D-3L dimensions in 18 countries.

**Country**	**EQ-5D-3L dimension**	**Sex**	**95% CI**		**Age^#^**	**95% CI**		**Educational level**	**95% CI**	
Belgium	Mobility	1.37	0.98	1.91	1.64	1.46	1.84	1.05	0.70	1.58
	Self-care	1.37	0.91	2.07	1.63	1.30	2.03	0.91	0.53	1.56
	Usual activities	1.47	1.13	1.91	1.50	1.33	1.68	1.17	0.83	1.65
	Pain/discomfort	1.31	1.05	1.63	1.31	1.22	1.42	1.19	0.88	1.63
	Anxiety/depression	1.63	1.04	2.55	1.05	0.93	1.20	1.04	0.60	1.82
China	Mobility	1.18	0.95	1.45	1.71	1.58	1.84	1.89	1.46	2.46
	Self-care	1.08	0.82	1.42	1.53	1.39	1.68	1.71	1.23	2.38
	Usual activities	1.20	0.97	1.48	1.55	1.44	1.67	2.47	1.89	3.23
	Pain/discomfort	1.67	1.43	1.95	1.55	1.47	1.63	1.54	1.29	1.83
	Anxiety/depression	1.19	1.01	1.40	1.23	1.17	1.30	2.36	1.95	2.86
Denmark	Mobility	1.25	1.12	1.38	1.41	1.37	1.45	1.82	1.62	2.04
	Self-care	1.25	1.02	1.53	1.51	1.42	1.59	1.89	1.49	2.40
	Usual activities	1.48	1.36	1.61	1.28	1.25	1.31	1.62	1.48	1.76
	Pain/discomfort	1.41	1.32	1.51	1.17	1.15	1.20	1.41	1.32	1.51
	Anxiety/depression	1.68	1.54	1.83	1.06	1.04	1.09	1.33	1.22	1.46
England	Mobility	1.22	1.11	1.35	1.65	1.59	1.70	2.17	1.95	2.41
	Self-care	1.19	1.02	1.40	1.47	1.40	1.55	2.33	1.95	2.79
	Usual activities	1.28	1.16	1.41	1.47	1.43	1.52	2.04	1.82	2.27
	Pain/discomfort	1.16	1.08	1.26	1.39	1.36	1.43	1.72	1.57	1.88
	Anxiety/depression	1.52	1.39	1.67	1.03	1.00	1.06	1.52	1.37	1.68
Finland	Mobility	1.04	0.91	1.18	2.17	2.06	2.28	1.89	1.65	2.16
	Self-care	0.96	0.80	1.15	2.24	2.08	2.41	1.78	1.46	2.17
	Usual activities	1.17	1.02	1.34	1.92	1.83	2.02	1.82	1.57	2.10
	Pain/discomfort	1.32	1.19	1.46	1.46	1.40	1.52	1.62	1.44	1.81
	Anxiety/Depression	1.26	1.08	1.46	1.16	1.10	1.22	1.50	1.28	1.77
France	Mobility	1.63	1.22	2.17	1.91	1.73	2.10	1.36	0.97	1.89
	Self-Care	0.94	0.59	1.49	1.68	1.43	1.99	1.51	0.86	2.65
	Usual activities	1.22	0.89	1.66	1.54	1.38	1.72	1.29	0.89	1.88
	Pain/Discomfort	1.19	0.98	1.44	1.39	1.30	1.48	1.20	0.97	1.49
	Anxiety/depression	1.16	0.90	1.49	1.01	0.93	1.09	0.93	0.71	1.22
Germany	Mobility	1.18	0.96	1.46	1.92	1.79	2.06	1.89	1.16	3.09
	Self-care	1.47	0.90	2.39	2.17	1.84	2.57	1.91	1.00	3.67
	Usual ACTIVITIES	1.22	0.93	1.59	1.69	1.54	1.86	1.96	1.25	3.08
	Pain/discomfort	1.36	1.15	1.60	1.38	1.28	1.48	1.59	0.99	2.56
	Anxiety/depression	1.43	0.98	2.07	1.04	0.90	1.19	1.79	0.82	3.91
Greece	Mobility	1.34	0.71	2.53	1.93	1.54	2.42	2.13	1.06	4.29
	Self-care	4.76	1.75	13.01	2.58	1.76	3.78	1.54	0.55	4.33
	Usual activities	1.95	0.93	4.11	2.52	1.86	3.40	2.22	0.96	5.14
	Pain/discomfort	1.83	1.04	3.20	1.59	1.32	1.92	3.03	1.62	5.68
	Anxiety/depression	1.27	0.66	2.43	1.19	0.96	1.47	3.79	1.71	8.36
Hungary	Mobility	1.17	1.00	1.37	1.80	1.71	1.89	2.00	1.70	2.35
	Self-care	0.84	0.66	1.08	1.84	1.69	2.00	2.61	2.00	3.40
	Usual activities	1.02	0.86	1.21	1.64	1.56	1.73	2.35	1.97	2.80
	Pain/discomfort	1.45	1.28	1.64	1.48	1.43	1.54	1.95	1.71	2.23
	Anxiety/depression	1.71	1.51	1.93	1.24	1.20	1.29	1.98	1.74	2.26
Italy	Mobility	1.44	1.15	1.79	2.25	2.07	2.45	1.78	1.30	2.43
	Self-care	1.94	1.39	2.70	2.16	1.88	2.48	1.81	1.12	2.91
	Usual activities	1.77	1.41	2.20	1.91	1.76	2.07	2.00	1.46	2.75
	Pain/discomfort	1.74	1.50	2.02	1.53	1.46	1.60	1.47	1.24	1.75
	Anxiety/depression	2.26	1.81	2.81	1.25	1.17	1.35	1.20	0.90	1.59
Korea	Mobility	2.40	1.37	4.22	1.66	1.29	2.15	3.56	1.81	7.03
	Self-care	6.53	0.77	55.11	3.52	1.44	8.64	3.34	0.31	35.97
	Usual activities	1.67	0.87	3.20	1.60	1.17	2.18	6.72	2.77	16.27
	Pain/discomfort	1.73	1.28	2.34	1.63	1.42	1.86	2.51	1.70	3.71
	Anxiety/depression	2.05	1.51	2.80	1.31	1.14	1.49	1.42	0.93	2.16
Netherlands	Mobility	1.60	1.12	2.29	1.53	1.37	1.70	1.38	0.95	2.01
	Self-care	2.93	1.60	5.39	1.36	1.07	1.72	1.08	0.55	2.13
	Usual activities	1.97	1.43	2.71	1.30	1.19	1.42	1.14	0.82	1.60
	Pain/discomfort	1.42	1.13	1.78	1.22	1.13	1.31	1.06	0.84	1.35
	Anxiety/depression	2.12	1.08	4.15	0.80	0.63	1.01	2.41	1.07	5.46
New	Mobility	1.04	0.77	1.40	1.75	1.58	1.93	1.26	0.92	1.73
Zealand	Self-care	0.77	0.45	1.33	1.76	1.46	2.13	1.28	0.73	2.25
	Usual activities	1.11	0.83	1.47	1.58	1.44	1.73	1.09	0.80	1.48
	Pain/discomfort	1.08	0.86	1.37	1.45	1.34	1.56	1.29	0.99	1.68
	Anxiety/depression	1.43	1.08	1.89	1.11	1.02	1.21	1.27	0.94	1.71
Slovenia	Mobility	0.70	0.48	1.02	1.95	1.72	2.20	4.48	2.64	7.58
	Self-care	0.87	0.54	1.39	1.67	1.45	1.93	3.89	2.30	6.58
	Usual activities	0.93	0.66	1.31	1.51	1.37	1.68	3.29	2.04	5.31
	Pain/discomfort	1.04	0.76	1.43	1.52	1.37	1.67	2.30	1.39	3.81
	Anxiety/depression	1.13	0.83	1.54	1.16	1.06	1.27	1.66	1.06	2.59
Spain	Mobility	1.61	1.30	2.00	1.91	1.78	2.06	1.46	1.10	1.96
	Self-care	2.02	1.36	3.01	1.79	1.58	2.03	2.12	1.20	3.74
	Usual activities	1.76	1.39	2.24	1.63	1.51	1.75	1.37	1.01	1.88
	Pain/discomfort	1.71	1.43	2.05	1.34	1.28	1.41	1.41	1.15	1.73
	Anxiety/depression	1.86	1.41	2.46	1.15	1.08	1.23	1.48	1.10	2.01
Sweden	Mobility	1.37	0.71	2.61	1.68	1.34	2.11	1.36	0.67	2.75
	Self-care	3.06	0.60	15.69	1.39	0.83	2.32	11.63	1.24	109.0
	Usual activities	0.97	0.51	1.87	1.27	1.03	1.57	1.58	0.77	3.26
	Pain/discomfort	1.11	0.77	1.62	1.26	1.12	1.42	2.05	1.33	3.16
	Anxiety/depression	1.74	1.16	2.63	0.94	0.83	1.07	1.41	0.87	2.28
Thailand*	Mobility	1.30	1.01	1.67	1.57	1.42	1.72	-	-	-
	Self-care	0.93	0.64	1.36	1.40	1.22	1.61	-	-	-
	Usual activities	0.97	0.75	1.24	1.22	1.11	1.34	-	-	-
	Pain/discomfort	1.37	1.09	1.71	1.31	1.20	1.43	-	-	-
	Anxiety/depression	1.44	1.17	1.79	1.14	1.05	1.23	-	-	-
United	Mobility	0.90	0.75	1.09	1.65	1.56	1.76	1.68	1.37	2.06
Kingdom	Self-care	0.80	0.57	1.13	1.45	1.30	1.62	1.85	1.26	2.71
	Usual activities	0.88	0.72	1.07	1.40	1.32	1.48	1.56	1.27	1.92
	Pain/discomfort	1.02	0.87	1.19	1.39	1.33	1.46	1.77	1.50	2.09
	Anxiety/depression	1.35	1.14	1.61	1.13	1.07	1.18	1.52	1.26	1.82
United States	Mobility	1.25	1.17	1.34	1.73	1.70	1.77	1.96	1.80	2.14
	Self-care	1.04	0.93	1.16	1.61	1.55	1.68	2.33	2.06	2.63
	Usual activities	1.43	1.35	1.52	1.54	1.51	1.57	1.84	1.69	2.01
	Pain/discomfort	1.30	1.24	1.37	1.46	1.43	1.48	1.45	1.35	1.57
	Anxiety/depression	1.49	1.42	1.57	1.12	1.10	1.14	1.42	1.33	1.51

* Education variable not available.

# Age was defined by 10-year age groups 18–24; 25–34; 35–44; 45–54; 55–64; 65–74; 75+ (reference group 18–24 years); sex was recorded as a categorical variable (reference group males); educational level in each country was categorized into low (equivalent to primary), medium (equivalent to secondary) and high (equivalent to levels above secondary education) and dichotomised (reference group medium/high).

Generally, each decade of age added higher likelihood for reported problems along the EQ-5D-3L dimensions. The only exception was anxiety/depression in the Netherlands and Sweden, where the odds decreased with age. In all other countries, anxiety/depression had increased odds with age, but among the 5 dimensions the mood dimension had the smallest odds ratio. Generally women had higher odds to report problems than men. However, exceptions included mobility, self-care, and usual activities in some countries. Sex-related odds ratios were highest for the self-care dimension in Korea (6.5), Greece (4.8), and in Sweden (3.1).

Educational level was also important in determining odds ratios after taking into account the impact of age and sex. The analysis showed widespread evidence that people with low educational level generally report more problems across the 5 dimensions of the EQ-5D-3L descriptive system in all countries. The only exceptions were individuals with low educational level reporting slightly lower level of likelihood for problems with self-care in Belgium and anxiety/depression in France. Generally, the highest likelihood for reporting problems among those with low educational level was observed in Korea, with odds ratios ranging from 1.4 to 6.7 across EQ-5D-3L dimensions in this country.

For the 7 countries with income data, income-related odds ratios are reported in Table 4. The vast majority of calculated income-related odds ratios were found to be >1, showing evidence for the widespread existence of income-related inequities in self-reported problems along the 5 dimensions of the EQ-5D-3L descriptive system in all countries. Income-related odds ratios were <1 only in three cases (pain/discomfort in Spain and anxiety/depression in France and The Netherlands) but none of these ratios were statistically significant.

**Table 4:** Logistic regression odds ratios for reporting any problems on each EQ-5D-3L dimension in 7 countries.

	**Belgium**		**France**		**Germany**		**Italy**		**Netherlands**		**Spain**		**US MEPS**	
	**Odds ratio**	**Std. Err**.	**Odds ratio**	**Std. Err**.	**Odds ratio**	**Std. Err**.	**Odds ratio**	**Std. Err**.	**Odds ratio**	**Std. Err**.	**Odds ratio**	**Std. Err**.	**Odds ratio**	**Std. Err**.
**Mobility**													
Sex	1.373	0.227	1.618	0.239	1.155	0.124	1.450	0.163	1.585	0.288	1.606	0.177	1.165	0.041
Age groups	1.645	0.094	1.912	0.094	1.925	0.066	2.297	0.102	1.530	0.085	1.917	0.073	1.748	0.018
Edu2	1.017	0.212	1.323	0.227	1.840	0.464	1.609	0.259	1.324	0.258	1.426	0.213	1.470	0.065
Income	1.066	0.035	1.045	0.070	1.122	0.059	1.185	0.066	1.093	0.081	1.075	0.056	1.361	0.020
_Cons	0.009	0.004	0.003	0.001	0.006	0.002	0.001	0.000	0.007	0.003	0.002	0.001	0.009	0.001
**Self care**													
Sex	1.379	0.293	0.909	0.215	1.418	0.353	1.962	0.333	2.869	0.903	2.016	0.408	0.917	0.052
Age groups	1.632	0.179	1.709	0.142	2.174	0.182	2.200	0.159	1.364	0.161	1.794	0.116	1.613	0.030
Edu2	0.876	0.239	1.351	0.390	1.883	0.632	1.614	0.397	0.996	0.360	2.055	0.604	1.613	0.114
Income	1.078	0.071	1.212	0.135	1.212	0.139	1.202	0.108	1.195	0.177	1.102	0.102	1.522	0.036
_Cons	0.003	0.002	0.002	0.001	0.000	0.000	0.000	0.000	0.001	0.001	0.000	0.000	0.002	0.000
**Usual activities**												
Sex	1.473	0.194	1.191	0.191	1.172	0.162	1.785	0.203	1.949	0.319	1.747	0.214	1.330	0.042
Age groups	1.503	0.086	1.556	0.084	1.695	0.079	1.948	0.082	1.303	0.060	1.636	0.061	1.559	0.015
Edu2	1.128	0.192	1.210	0.232	1.899	0.440	1.791	0.293	1.102	0.193	1.288	0.205	1.350	0.063
Income	1.087	0.040	1.124	0.085	1.200	0.095	1.215	0.069	1.081	0.072	1.187	0.063	1.388	0.019
_Cons	0.010	0.003	0.008	0.003	0.005	0.002	0.001	0.000	0.013	0.005	0.003	0.001	0.012	0.001
**Pain/discomfort**												
Sex	1.298	0.143	1.163	0.115	1.336	0.117	1.741	0.132	1.405	0.162	1.716	0.157	1.252	0.031
Age groups	1.319	0.049	1.412	0.045	1.382	0.049	1.538	0.037	1.221	0.045	1.341	0.034	1.470	0.013
Edu2	1.167	0.180	1.116	0.125	1.553	0.369	1.420	0.127	1.029	0.130	1.423	0.150	1.207	0.048
Income	1.052	0.026	1.150	0.055	1.097	0.053	1.069	0.041	1.073	0.057	0.979	0.043	1.211	0.013
_Cons	0.073	0.019	0.075	0.018	0.052	0.012	0.018	0.004	0.111	0.030	0.029	0.007	0.092	0.006
**Anxiety/depression**												
Sex	1.605	0.364	1.161	0.150	1.404	0.271	2.257	0.253	2.150	0.743	1.850	0.262	1.415	0.037
Age groups	1.065	0.068	1.004	0.042	1.040	0.074	1.257	0.048	0.796	0.098	1.155	0.039	1.127	0.011
Edu2	0.987	0.287	0.941	0.136	1.755	0.712	1.185	0.176	2.521	1.091	1.412	0.218	1.116	0.037
Income	1.143	0.053	0.974	0.065	1.088	0.095	1.023	0.063	0.897	0.137	1.130	0.071	1.285	0.014
_Cons	0.015	0.007	0.149	0.045	0.018	0.008	0.009	0.002	0.021	0.015	0.009	0.003	0.081	0.005

Shaded fields in the table highlight the income related odds ratios. Values greater than 1 indicate the existence of income-related inequities in reported problems along the 5 dimensions of the EQ-5D-3L descriptive system.

Across the 5 dimensions of the EQ-5D-3L descriptive system, the current analysis did not provide clear evidence that problems reported in any particular dimension would drive income-related inequity across all countries. Income-related odds ratios were highest for self-care in 4 of the 7 countries, while they were highest for usual activities in 3 countries. The income-related odds ratio was highest for anxiety/depression in 1 country (Belgium). The top three highest income-related odds ratios also included mobility and pain/discomfort in some countries. Three cases of income-related odds ratios below the value of 1 were found in anxiety/depression and in pain/discomfort.

The US had the highest income-related odds ratios along all 5 dimensions of the EQ-5D-3L descriptive system compared to all other countries in the analysis. The income-related odds ratios in the US varied from 1.21 for reporting pain/discomfort to 1.52 for reporting problems with self-care. This means that belonging to a lower income category is associated with a substantially increased likelihood of experiencing a health problem, after adjusting for other socio-demographic variables.

### Concentration index results

Results of the concentration index analysis of the 17 countries (not including Finland) are shown in Table 5 (by socio-demographic indicators), Table 6 (by 5 dimensions of the EQ-5D-3L descriptive system), and Table 7 (by socio-demographic indicators and the 5 dimensions of the EQ-5D-3L descriptive system). Findings suggest that the level of differences in self-assessed overall EQ VAS score and the health concentration pattern by EQ-5D-3L dimension differed across countries. In terms of the overall level of health concentration, Korea, Denmark, and China presented the lowest concentration (0.090, 0.094, and 0.095 respectively) while Spain and Hungary the highest concentration of health (0.173 and 0.157, respectively). Differences were discerned in the extent to which the socio-demographic indicators and the the 5 dimensions of the EQ-5D-3L descriptive system explained concentration in overall self-assessed health measured on the EQ VAS (Tables 5, 6).

**Table 5:** Health concentration index based on overall self-assessed health measured on the EQ VAS as explained by socio-demographic indicators.

**Country**	Concentration index*	**Socio-demographic indicator (absolut % and relative %)**			
		**Explained share^#^**	**Sex**	**Age**	**Educational level**
Belgium	0.126	7.9	0.2	7.7	0.0
		100.0	1.9	97.8	0.3
China	0.095	21.7	0.1	12.9	8.6
		100.0	0.6	59.6	39.7
Denmark	0.094	7.0	0.0	4.3	2.7
		100.0	0.0	61.2	38.8
France	0.132	12.8	0.0	11.9	0.9
		100.0	0.0	93.3	6.7
Germany	0.131	17.8	0.1	16.9	0.8
		100.0	0.5	95.2	4.3
Greece	0.125	16.5	0.5	12.7	3.3
		100.0	2.9	77.2	19.9
Hungary	0.157	24.4	0.4	19.6	4.4
		100.0	1.5	80.2	18.2
Italy	0.133	19.0	0.7	17.6	0.7
		100.0	3.6	92.5	3.9
Korea	0.090	3.0	0.2	0.0	2.8
		100.0	6.1	0.0	93.9
Netherlands	0.104	4.7	0.4	3.7	0.6
		100.0	8.8	78.1	13.0
New Zealand	0.103	2.4	0.1	2.1	0.2
		100.0	2.9	86.8	10.3
Slovenia	0.136	27.6	0.3	15.9	11.4
		100.0	1.1	57.7	41.2
Spain	0.173	7.5	0.5	6.7	0.4
		100.0	6.4	88.8	4.9
Sweden	0.103	4.0	0.1	1.6	2.3
		100.0	2.7	40.1	57.2
Thailand∧	0.108	0.9	0.2	0.7	-
		100.0	21.7	78.3	-
United Kingdom	0.110	9.0	0.0	5.9	3.1
		100.0	0.1	65.6	34.3
United States	0.112	9.3	0.3	7.6	1.4
		100.0	3.7	81.5	14.8

* *P* < 0.05 in all countries. ∧Educational level variable is not available in Thailand.

# Percentages in the first row for each country show absolute value, i.e., proportion of the health concentration index explained by each indicator. Percentages in the second row for each country show relative value, i.e., relative share of each indicator in the explained portion of the health concentration index.

**Table 6:** Health concentration index based on overall self-assessed health measured on the EQ VAS as explained by 5 dimensions of the EQ-5D-3L descriptive system.

**Country**	Concentration index*	**EQ-5D dimensions**					
		**(absolut %)**					
		**(relative %)**					
		**Explained share^#^**	**Mobility**	**Self-care**	**Usual activities**	**Pain/discomfort**	**Anxiety/depression**
Belgium	0.126	24.9	5.2	3.5	8.2	4.7	3.4
		100.0	20.9	13.9	32.9	18.8	13.5
China	0.095	24.4	3.1	0.2	2.8	9.5	8.9
		100.0	12.5	0.8	11.4	38.9	36.4
Denmark	0.094	36.5	7.0	2.6	12.7	8.4	5.8
		100.0	19.3	7.2	34.7	23.0	15.8
France	0.132	24.2	5.4	3.0	4.6	7.4	3.7
		100.0	22.5	12.5	19.1	30.5	15.4
Germany	0.131	34.6	11.3	1.6	9.1	9.3	3.3
		100.0	32.8	4.6	26.4	26.8	9.4
Greece	0.125	54.3	20.4	0.2	16.5	11.7	5.6
		100.0	37.5	0.4	30.3	21.5	10.3
Hungary	0.157	46.3	9.0	2.9	6.8	18.3	9.3
		100.0	19.5	6.3	14.7	39.5	20.0
Italy	0.133	35.2	7.7	2.7	9.2	10.8	4.8
		100.0	21.8	7.6	26.2	30.7	13.7
Korea	0.090	16.8	0.3	0.3	2.8	8.3	5.1
		100.0	1.8	2.0	16.6	49.6	30.1
Netherlands	0.104	30.7	6.5	0.4	14.7	8.3	0.9
		100.0	21.1	1.2	48.0	26.9	2.8
New Zealand	0.103	37.4	7.3	8.1	10.6	5.0	6.4
		100.0	19.5	21.7	28.3	13.4	17.2
Slovenia	0.136	54.3	13.0	9.3	14.6	11.9	5.5
		100.0	24.0	17.1	26.8	21.9	10.1
Spain	0.173	21.5	5.1	0.6	5.5	7.7	2.7
		100.0	23.5	2.9	25.5	35.7	12.5
Sweden	0.103	43.9	2.6	2.6	9.6	16.6	12.6
		100.0	5.9	5.9	21.8	37.7	28.6
Thailand	0.108	14.6	1.5	0.0	2.2	8.4	2.7
		100.0	10.2	0.0	15.1	57.2	18.5
United Kingdom	0.110	35.0	7.1	1.6	9.7	9.6	7.0
		100.0	20.3	4.5	27.7	27.4	20.1
United States	0.112	42.6	8.2	4.3	13.8	7.8	8.5
		100.0	19.3	10.1	32.5	18.2	19.9

* *P* < 0.05 in all countries.

# Percentages in the first row for each country show absolute value, i.e., proportion of the health concentration index explained by each indicator. Percentages in the second row for each country show relative value, i.e., relative share of each indicator in the explained portion of the health concentration index.

**Table 7:** Health concentration index based on overall self-assessed health measured on the EQ VAS as explained by socio-demographic indicators and the 5 dimensions of the EQ-5D-3L descriptive system.

	Inequality index*	**Explained share^#^**	**Sex**	**Age**	**Educational level**	**Mobility**	**Self-care**	**Usual activities**	**Pain/ discomfort**	**Anxiety/ depression**
Belgium	0.126	26.8	0.0	3.8	0.0	4.3	3.4	7.8	4.2	3.4
		100.0	0.0	14.0	0.0	16.1	12.6	29.0	15.5	12.7
China	0.095	36.6	0.1	11.5	4.1	2.3	0.2	2.4	7.4	8.6
		100.0	0.3	31.3	11.3	6.2	0.7	6.5	20.3	23.4
Denmark	0.094	38.4	0.1	1.6	1.7	7.1	2.4	12.0	8.1	5.5
		100.0	0.1	4.2	4.3	18.5	6.3	31.1	21.1	14.4
France	0.132	28.6	0.0	7.0	0.7	3.5	2.9	4.5	6.1	4.0
		100.0	0.0	24.5	2.4	12.1	10.0	15.7	21.4	13.8
Germany	0.131	39.4	0.0	9.0	0.3	8.3	1.3	8.7	8.5	3.4
		100.0	0.0	22.9	0.7	21.1	3.2	22.0	21.4	8.7
Greece	0.126	55.2	0.1	2.6	0.3	20.4	0.0	15.4	10.4	6.0
		100.0	0.1	4.7	0.5	37.1	0.0	27.9	18.9	10.8
Hungary	0.157	50.6	0.0	9.4	1.2	6.6	2.4	5.9	15.8	9.2
		100.0	0.0	18.7	2.3	13.1	4.8	11.8	31.3	18.1
Italy	0.133	38.8	0.0	8.0	0.1	5.6	2.5	8.7	9.1	4.8
		100.0	0.0	20.5	0.2	14.5	6.4	22.4	23.5	12.4
Korea	0.090	19.3	0.0	0.0	1.5	0.4	0.4	2.7	9.0	5.3
		100.0	0.0	0.0	7.9	2.1	2.0	14.0	46.6	27.4
Netherlands	0.104	31.4	0.0	1.4	0.4	5.7	0.4	14.7	8.0	0.9
		100.0	0.0	4.4	1.2	18.2	1.2	46.7	25.4	2.8
New Zealand	0.103	38.9	0.1	0.0	0.1	8.1	8.1	10.6	5.3	6.5
		100.0	0.2	0.0	0.3	20.8	20.9	27.3	13.8	16.8
Slovenia	0.136	57.7	0.3	4.1	6.2	8.8	7.4	13.9	11.3	5.7
		100.0	0.5	7.2	10.7	15.3	12.7	24.2	19.6	9.9
Spain	0.173	22.8	0.1	2.8	0.1	4.0	0.6	5.2	7.2	2.7
		100.0	0.6	12.4	0.6	17.5	2.5	22.9	31.6	12.0
Sweden	0.104	44.4	0.0	0.4	0.3	2.4	2.5	9.7	16.3	12.8
		100.0	0.1	0.9	0.7	5.4	5.6	21.9	36.7	28.8
Thailand+	0.108	15.4	0.4	0.2	-	1.5	0.0	2.1	8.4	2.9
		100.0	2.3	1.0	-	9.5	0.0	13.9	54.7	18.6
United Kingdom	0.110	35.9	0.0	1.1	1.3	6.3	1.5	9.8	8.9	6.9
		100.0	0.0	3.0	3.7	17.7	4.3	27.2	24.7	19.3
United States	0.112	43.1	0.0	1.1	0.5	7.6	4.0	13.9	7.4	8.5
		100.0	0.0	2.6	1.2	17.5	9.4	32.2	17.3	19.8

* *P* < 0.05 in all countries. ^+^Education variable is not available in Thailand.

# Percentages in the first row for each country show absolute value, i.e., proportion of the health concentration index explained by each indicator. Percentages in the second row for each country show relative value, i.e., relative share of each indicator in the explained portion of the health concentration index.

When both socio-demographic indicators and the 5 dimensions of the EQ-5D-3L descriptive system were included as explanatory variables for overall self-assessed health measured on the EQ VAS, the statistical model showed that 15.4–57.7% of the concentration index was explained by these variables included in this model. Within the socio-demographic indicators, sex played the smallest role in explaining overall concentration of self-assessed EQ VAS health (0–2.3% in relative terms within the explained share). Age was generally the most important determinant (0–31.3%). After taking into account age and sex, educational level played a variable role in explaining health concentration in each country, from 0% in Belgium to 11.3% in China (Table 7).

The decomposition analysis that combined both the socio-demographic variables and reported problems along the 5 dimensions of the EQ-5D-3L descriptive system revealed that usual activities became the strongest contributor to inequalities in overall self-assessed health measured on the EQ VAS in the majority of countries (*n* = 9) followed by pain/discomfort (Table 7).

### Income-related inequalities in overall health measured by EQ VAS

Generally, weak association was found between income and EQ VAS score in the European countries, while stronger relationship between income level and EQ VAS score was seen in the US data as reflected by the following correlation values: Belgium 0.016; France −0.025; Germany 0.042; Italy −0.022; Netherlands 0.066; Spain 0.036; and the US 0.155.

The income-related health (based on EQ VAS) concentration index was not statistically significantly different from zero in three European countries, including France, Italy, and Spain. Among the 4 other countries where statistically significant income-related inequalities were shown, the highest concentration index was observed in the US (CI = 0.0234, *p* = 0.0000). In these countries, socio-demographic indicators explained 2.4–21.7% of the income-related inequalities in overall self-assessed health measured on the EQ VAS. Among these indicators, educational level and sex played relatively bigger role compared to age (Table 8).

**Table 8:** Income-related health concentration index based on overall self-assessed health measured on the EQ VAS and decomposition analysis in 7 countries.

**Country**	**Income related health concentration index^#^**	**Socio-demographic indicators** ^**+**^				**5 dimensions of the EQ-5D-3L descriptive system**					
		**Explained share %**	**Sex%**	**Age %**	**Educational level %**	**Explained share %**	**Mobility%**	**Self-care %**	**Usual activities%**	**Pain/ discomfort%**	**Anxiety/ depression%**
Belgium	0.0094 (*P =* 0.002)	2.4	93.3	0.0	6.7	44.1	13.3%	12.9	32.4	8.9	32.5
France	0.0029* (*P =* 0.299)										
Germany	0.0078 (*P =* 0.001)	8.0	41.3	0.0	58.7	54.6	25.9	7.2	33.3	23.9	9.7
Italy	0.0024* (*P =* 0.260)										
Netherlands	0.0133 (*P =* 0.000)	13.8	36.2	5.6	58.3	26.4	27.2	2.3	42.9	26.9	0.8
Spain	0.0014* (*P =* 0.593										
United States	0.0234 (*P =* 0.000)	21.7	22.2	−29.5	107.3	74.9	20.2	14.8	33.8	11.6	19.5

* The concentration index was not statistically significantly different from zero in France, Italy, and Spain.

^+^Percentages in the first coloun under the socio-demographic and the 5 dimensions of the EQ-5D-3L descriptive system show the proportion of the health concentration index explained by each model. Percentages under each variable then show relative contribution to the explained portion of the health concentration index.

# The income-related health concentration index based on overall self-assessed health measured on the EQ VAS was the highest in the US with a value of 0.0234. Socio-demographic indicators explained 21.7% of the concentration index in the US. In total 74.9% of the health concentration index was explained by reported problems along the 5 dimensions of the EQ-5D-3L descriptive system in the US.

Reporting problems along the 5 dimensions of the EQ-5D-3L descriptive system explained a large part (26.4–74.9%) of income-related inequalities in overall self-assessed health measured on the EQ VAS across the 4 countries. Problems with usual activities was the greatest contributor to overall inequalities in all four countries, although in Belgium anxiety/depression was an equally important contributor (Table 8).

Additional analyses that were based on equivalent income measurement criteria yielded similar results. Problems with usual activities were still a leading contributor to inequalities in overall self-assessed health measured on the EQ VAS in most countries. However, the relative importance of pain/discomfort was greater than usual activities in France, while mobility was the highest contributor in Germany.

## Discussion

This study is one of the first international analyses on a large scale that demonstrates the measurement of socio-demographic differences in self-assessed overall health among general populations using an internationally validated HRQoL instrument, the EQ-5D-3L. Our analyses provide researchers with a better understanding of how the EQ-5D-3L helps to explore and monitor variations and inequalities in health along a multi-dimensional measure as opposed to traditionally used single dimension or overall measures of health. The EQ-5D-3L instrument was shown to be a powerful measure in the socio-demographic analysis of self-reported health using various methods, including both the odds ratios that focus on comparing groups and concentration indices that consider the entire distribution within populations.

Results showed that significant differences exist in self-reported health as measured by the EQ-5D-3L across socio-demographic groups in each country involved in the study. Both the analysis of odds ratios and concentration indices showed that age is the most important predictor of experiencing lower EQ VAS score and problems on mobility, self-care, usual activities, and pain/discomfort in all countries. Sex does play an additional role, although its role is much smaller.

Having attained at least a medium educational level, adjusted for age and sex, translated into lower odds of reporting problems on any dimension of EQ-5D-3L in almost all surveyed countries. However, this relationship seemed to possess some country-specific traits that deserve the attention of policy makers. Evidence on differences by educational level in self-reported health is an important finding as such differences in health are often seen as unfair and potentially avoidable; that is, an inequity.

Furthermore, results from this research also suggest significant differences in self-reported health as measured by reported problems along the 5 dimensions of the EQ-5D-3L descriptive system by the individual's income level after adjusting for age, sex, and educational level. This is an important finding as income-related differences in health are also often seen as unfair and avoidable. These results, or potential similar findings in future studies, may inform policy-makers how to best design programs with respect to targeting groups of low income levels vs. focusing on groups with low educational level. However, the concentration index method did not detect statistically significant income-related inequalities in overall self-assessed health measured on the EQ VAS in 3 of the European countries, suggesting a need for further investigation.

Among countries, there was a clear difference between European countries and the US, with US data suggesting greater income-related health inequalities in all aspects of health measured by the EQ-5D-3L. This finding raises further research questions whether the cause may be linked to larger income inequalities in the US or due to health care system characteristics related to lower coverage and access to care among lower income groups in the US.

The decomposition analysis of the concentration index provided a unique insight into the role of each individual EQ-5D-3L dimension in explaining overall concentration of self-assessed health. This analysis, in particular, highlighted the widespread importance of problems with usual activities and pain/discomfort in explaining distribution of self-assessed overall health as measured by the EQ VAS. For problems with anxiety/depression, age did not show the same pattern as in the other dimensions, reflecting that problems in the mood dimension were present also in younger age groups. This finding is an important information for policy makers.

The strength of our study is to show the potential use of the EQ-5D-3L in examining socio-demographic differences in health in different countries. Our results are in line with previous findings using the one-dimension self-rated health question (4) showing variation in inequalities in health associated with socio-economic status across European countries. The successful application of the EQ-5D (both 3L or 5L) in socio-demographic analyses in selected countries and regions were also confirmed by several other recent studies (36–39), and in vulnerable groups like homeless persons (40). Specifically, Sronk et al. showed that among three European countries the EQ-5D-5L was able to detect education level related inequalities in health, however, differences varied across countries and some of the impact was canceled out when controlling for chronic conditions and ability to work. Comparability of these findings with our study is limited by differences in stratifying across low, medium, and high education levels as opposed to focus on low education level in our study (36). Regarding education and income level, a Swedish study by Teni et al. (37) confirmed our finding that the EQ-5D-5L was able to detect socioeconomic gradients along these variables. This was the case even after taking into account health related behaviors and conditions (37). A study from five Caribbean countries used odds ratios and concentration indices to detect education and income related inequalities with the EQ-5D-5L and also found significant inequalities that varied across countries (38). Studies using the EQ-5D-3L provided similar evidence of detecting income related health inequalities in low income communities (39), and high reported problems among homeless people (40).

A limitation of our analysis was that socio-demogrpahic indicators were examined alongside 4 key variables. While age, sex, education, and income were shown to be important stratifiers, additional social characteristics, such as occupation, family status, race, among many other characteritics, can be also relevant to explore in future research with broader availability of data. Recent studies using single datasets have indeed explored a broader list of socio-demogrpahic and disease related variables in inequality analyses. Important social stratifyer variables included employment and occupational status, region and type of location (rural and urban), ethnicity, family and living status, loss in health insurance and access to care (38, 41, 42). Teni et al. also pointed out that the inclusion of variables on health related behaviors, diseases diagnosed by a physician, self-reported conditions, and BMI levels can also be important in inequality analyses as they can help to show the extent to which diferences between socioeconomic groups are explained by diferences in health-related behaviors (37). While the inclusion of all relavent socio-economic variables can enhance inequality analyses, international analyses of existing EQ-5D data are limited by the lack of availability of standard socio-economic variables across datasets and countries. This suggests the need for further harmonization of data collection methodology in future EQ-5D population studies.

Our international analysis lacked the use of the EQ-5D index, which is another frequently used measure of the instrument. The reason for this is that the EQ-5D index incorporates value sets that reflect preferences of populations regarding valuation of health states that vary across countries. As such, the use of EQ-5D index as a measure would add complexity to international analyses in terms of separating the impact of socio-demographic indicators from the impact of how people value health across different countries and cultures. However, the EQ-5D index (based on 3L or 5L data) as a single measure have been used in socio-demographic analysis within individual countries. For example, Arrospide et al. conducted inequality analysis using the EQ-5D-5L index as their main measure (43).

Finally, it has to be noted that the results from our analysis should not be used for ranking countries in terms of health inequality in their populations. Neither was the analysis designed to account for potential differences in demographic or other sample characteristics across countries. Each country should consider the results within the light of their own social and health care context. A shortcoming of our study is related to the large time interval for the surveys included in our analysis which reduce substantially the direct policy implications when data are collected years ago. However, results from past years are important in establishing baseline data on socio-demographic indicators of self-reported health against which later data can be compared, and policies assessed.

Indeed, several countries have started to use EQ-5D in monitoring population health. Adding the measurements of inequity can be important in tracking trends and evaluating policies. With the increased use of the EQ-5D-5L version of the EQ-5D in population health studies, it will be important to confirm if methods to measure inequalities used in this analysis based on binary measurement of EQ-5D-3L problems (no problem vs. any problem) would be comparable with data based on the EQ-5D-5L version.

## Conclusion

These analyses show that HRQoL instruments, such as the EQ-5D-3L, are a useful tool to explore socio-demographic differences in health by HRQoL dimensions or domains. Analytic methods, that focus on comparing groups, such as by odds ratios, and those that assess the entire population, such as concentration indices, are both informative when applied to EQ-5D-3L general population survey data. This study also demonstrated that socio-demographic differences in self-assessed overall health measured by the EQ VAS exist across many countries. The health concentration pattern of each country deserves policy attention to promote greater equity in health. This study, in particular, highlighted the importance of problems with usual activities and pain/discomfort in explaining concentration of self-assessed overall health measured by the EQ VAS. More research is warranted in general population surveys using HRQoL measures to study and monitor socio-economic differences in self-reported health.

## Data availability statement

The datasets presented in this article are not readily available because they were specifically provided only to the EuroQol Group for public health research purposes. The authors, as members of the EuroQol Group, conducted a secondary analysis of the data that were provided by various investigators/organizations. Requests to access the datasets should be directed to the corresponding authors.

## Author contributions

AS and MJ designed the analysis, interpreted results, and wrote the manuscript. MJ and JR-G analyzed the data. JC and KB interpreted results and contributed to the manuscript. All authors contributed to the article and approved the submitted version.

## Footnotes

^1^Source: OECD 2017. Available online at: https://www.oecd.org/economy/growth/OECD-Note-EquivalenceScales.pdf Note: The traditionally used “Old OECD Scale” applied a different scale of 1, 1.7, 2.2, 2.7, and 3.2 for household size of 1, 2, 3, 4, and 5 members, respectively.
