# Supplementary material for: Socio-demographic indicators of self-reported health based on EQ-5D-3L: A cross-country analysis of population surveys from 18 countries
Source: Front Public Health. 2023 Jan 6;10:959252. doi: 10.3389/fpubh.2022.959252 (PMC9853521; doi:10.3389/fpubh.2022.959252)
Supplement: Supplementary file 1 [file Table_1.DOC]

**Appendix A**

**Computation of concentration indices**

*A1. Computation of health concentration indices – all countries (except Finland)*

The health concentration index for overall self-reported health, as measured by the EQ VAS, was computed by the convenient regression model as proposed by Kakwani et al. (1997):

$$\frac{2\sigma_{R}^{2}}{\overline{EQVAS}}EQVAS_{i}=\alpha_{i}+\gamma_{k}R_{i}+\varepsilon_{i}$$

where *R_i_* is the relative fractional rank of the *i*^th^ individual (ranked by the individual’s EQVAS score), and γ_k_ is the estimated concentration index, while ɛ is the random error.

For the purposes of the decomposition analysis, the same estimation is used for all explanatory variables (by replacing EQ VAS score with the explanatory variable in the equation and also using this variable for ranking purposes).

The total health concentration index can be written as the weighted sum of the concentration indices of the explanatory variables and the generalized concentration index of ε:

$$\hat{C}=\sum_{k} \hat{\eta}_{k}\hat{C}_{xk}+G\hat{C}_{\varepsilon}$$

where the weights are equal to the elasticities of EQ VAS score with respect to each explanatory variable in the model:

$$\hat{\eta}_{k}=\hat{\gamma}_{k}\bar{x}_{k}/\overline{EQVAS}$$

where $\bar{x}_{k}$ (the mean of *x_k_* explanatory variables: age, sex, educationalal level, prevalence of EQ-5D-3L problems in each dimension) is multiplied by the coefficients for each explanatory variable that are taken from the linear regression model to explain EQ VAS score:

$EQ-VAS=\alpha+\sum_{k} \gamma_{k}\chi_{ik}+\varepsilon_{i}$.

*A2. Computation of income-related health concentration indices – 7 countries (Belgium, France, Germany, Italy, Netherlands, Spain, and the United States)*

The concentration index for overall self-reported health, as measured by the EQ-VAS, was computed by the convenient regression model as proposed by Kakwani et al. (1997)[[1]](#footnote-2):

$$\frac{2\sigma_{R}^{2}}{\overline{EQVAS}}EQVAS_{i}=\alpha_{i}+\gamma_{k}R_{i}+\varepsilon_{i}$$

where *R_i_* is the relative fractional rank of the i^th^ individual (ranked by the individual’s *income* level), and γ_k_ is the estimated concentration index.

For the purposes of the decomposition analysis, the same estimation is used for all explanatory variables (by replacing EQ VAS score with the explanatory variable in the equation).

The total health concentration index can be written as the weighted sum of the concentration indices of the explanatory variables and the generalized concentration index of

$$\hat{C}=\sum_{k} \hat{\eta}_{k}\hat{C}_{xk}+G\hat{C}_{\varepsilon}$$

where the weights are equal to the elasticities of EQ VAS score with respect to each explanatory variable in the model:

$$\hat{\eta}_{k}=\hat{\gamma}_{k}\bar{x}_{k}/\overline{EQVAS}$$

where (the mean of x_k_ explanatory variables: age, sex, educational level, prevalence of EQ-5D problems in each dimension) is multiplied by the coefficients for each explanatory variable that are taken from the linear regression model to explain EQ VAS score:

.

1. Kakwani NC, Wagstaff A, Doorslaer E. Socioeconomic inequalities in health: measurement, computation, and statistical inference. Journal of Econometrics 1997; 77:87-103. [↑](#footnote-ref-2)
